# COVID-19 and Person-Centered Care: Lessons Learned Through a Statewide Program for Nursing Homes

**DOI:** 10.1093/geroni/igab046.1041

**Published:** 2021-12-17

**Authors:** Migette Kaup, Laci Cornelison

**Affiliations:** Kansas State University, Manhattan, Kansas, United States

## Abstract

Frail elders in nursing homes are the highest risk group for developing complications of COVID-19. This lead to a response from CMS and state regulators that was heavily focused on protection and safety through segregation and infection control. The purpose of this study was to gather the narrative of this pandemic response and understand the impact on person-centered care and be able to address provider needs in real-time. This qualitative method focused on nursing home providers who are a part of PEAK 2.0, a Medicaid pay-for-performance program in Kansas. Interviews with nursing home staff (n=168) revealed two critical themes of need; mandated responses disregarded elders’ autonomy and self-determination in decision making, and infection control strategies required new approaches to facets of resident care that still maintained dignity. This data, along with COVID-19 guidance were then used to inform feasible resource development and education to maintain PCC practices during the pandemic.

